# Case Report: Adrenal Epithelial Cyst in an 11-Year-Old Leptailurus Serval

**DOI:** 10.3389/fvets.2022.897469

**Published:** 2022-06-06

**Authors:** Sacha L. Devereux, Wendy I. Baltzer, Susan A. Piripi, Mark C. Owen

**Affiliations:** ^1^Small Animal Surgery Department, School of Veterinary Science, Massey University Veterinary Teaching Hospital, Massey University, Palmerston North, New Zealand; ^2^Small Animal Surgery Department, University Veterinary Teaching Hospital, Sydney, NSW, Australia; ^3^IDEXX Laboratories, Clinical Pathology, School of Veterinary Science Complex, Massey University, Palmerston North, New Zealand; ^4^Radiology Department, School of Veterinary Science, Massey University Veterinary Teaching Hospital, Massey University, Palmerston North, New Zealand

**Keywords:** adrenal cyst, adrenal, serval, case report, epithelial cyst

## Abstract

**Case Description:**

A serval (Leptailurus serval) presented for progressive enlargement of the right adrenal gland, which had been found incidentally on abdominal ultrasound 2 years previously and upon subsequent ultrasound examinations enlarged progressively from 1.26 to 1.43 cm.

**Clinical Findings:**

Clinical signs had not been recorded by the zookeeper, however, progressive weight gain and lethargy were reported. Computed tomography (CT) confirmed the presence of a right caudal pole adrenal mass measuring 1.8 cm.

**Treatment and Outcome:**

The right adrenal with associated mass was surgically resected *via* a ventral midline laparotomy that included the resection of the right phrenicoabdominal vein in association with the mass. Histopathological examination identified the mass as an epithelial cyst. Chromogranin A staining for a possible pheochromocytoma was performed but was negative. The serval recovered at the zoo without complication and has been on display for 18 months.

**Clinical Relevance:**

Epithelial adrenal cysts have not been previously reported in felids. An adrenal cyst should be included on the differential list for any animal with an enlarged, slowly growing adrenal gland mass with non-specific clinical signs.

## Introduction

Adrenal cysts are rare and have not been reported in a feline to the authors' knowledge. As adrenal cysts are uncommonly reported in humans, there is no current consensus regarding the best management and treatment (1). Multiple options, such as surgical excision (2–4), percutaneous fine needle aspiration (2, 5), cystogram (6, 7), and marsupialization (8), for diagnosis and treatment of adrenal cysts, have been reported in the human literature. This case report is the first to describe the surgical treatment and outcome of a feline with a confirmed epithelial cyst.

## Case Description

**Figure 1:**
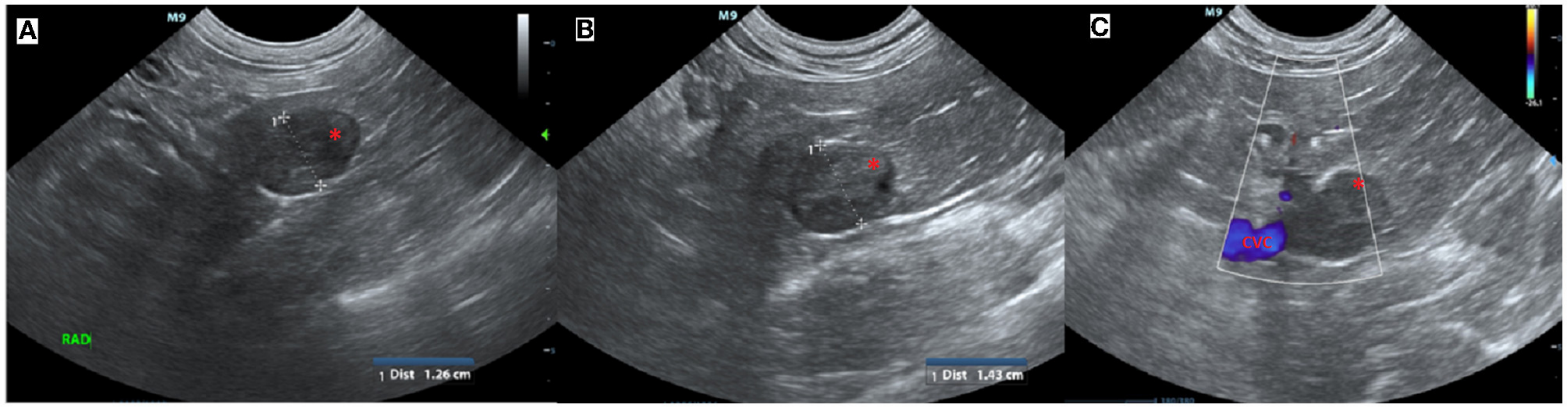
Ultrasound images of the right adrenal mass. **(A)** Initial ultrasound scan showing enlargement of the right adrenal gland (*). **(B)** A subsequent ultrasound scan approximately 11 months later showed progressive enlargement (calipers) of the right adrenal gland (*) and **(C)** in close association with the caudal vena cava (CVC, blue) but no evidence of invasion.

An 11-year-old female serval (Leptailurus serval) from Wellington Zoo was presented for a right adrenal mass, which had been progressively enlarging over the previous several months. The mass was initially found incidentally on abdominal ultrasound approximately 2 years previously while undergoing evaluation following a foreign body small intestinal obstruction (wood chip mulch). At initial diagnosis, the right adrenal gland was measured as 1.26 cm in comparison to the left adrenal gland, which measured 0.5 cm (Figure 1).

The zookeeper did not report any clinical signs other than lethargy and progressive weight gain over the last 2 years. Recovery following wood chip foreign body removal *via* small intestinal enterotomy at exploratory laparotomy surgery 2 years previously was reported by the zookeeper as uneventful, and no further gastrointestinal signs were noted.

Successive abdominal ultrasounds indicated that the adrenal mass was expanding without evidence of invasion into the surrounding caudal vena cava or renal vasculature. The mass was encroaching on the right phrenicoabdominal vein but invasion into the vein could not be determined using ultrasound, and computed tomographic evaluation was recommended (Figure 1).

### Diagnostic Assessment

Physical examination on the presentation for computed tomographic evaluation was limited due to the felid's fractious demeanor. While under general anesthesia, vital parameters, such as pre-operative blood pressure (150/100, MAP 115), heart rate (80 bpm), respiratory rate (20 bpm), and thoracic auscultation, were within normal limits for felid species under general anesthesia, however, her body condition score was 9/9. A blood sample was collected and found incompatible on major and minor cross-match with two donor domestic cats.

**Figure 2:**
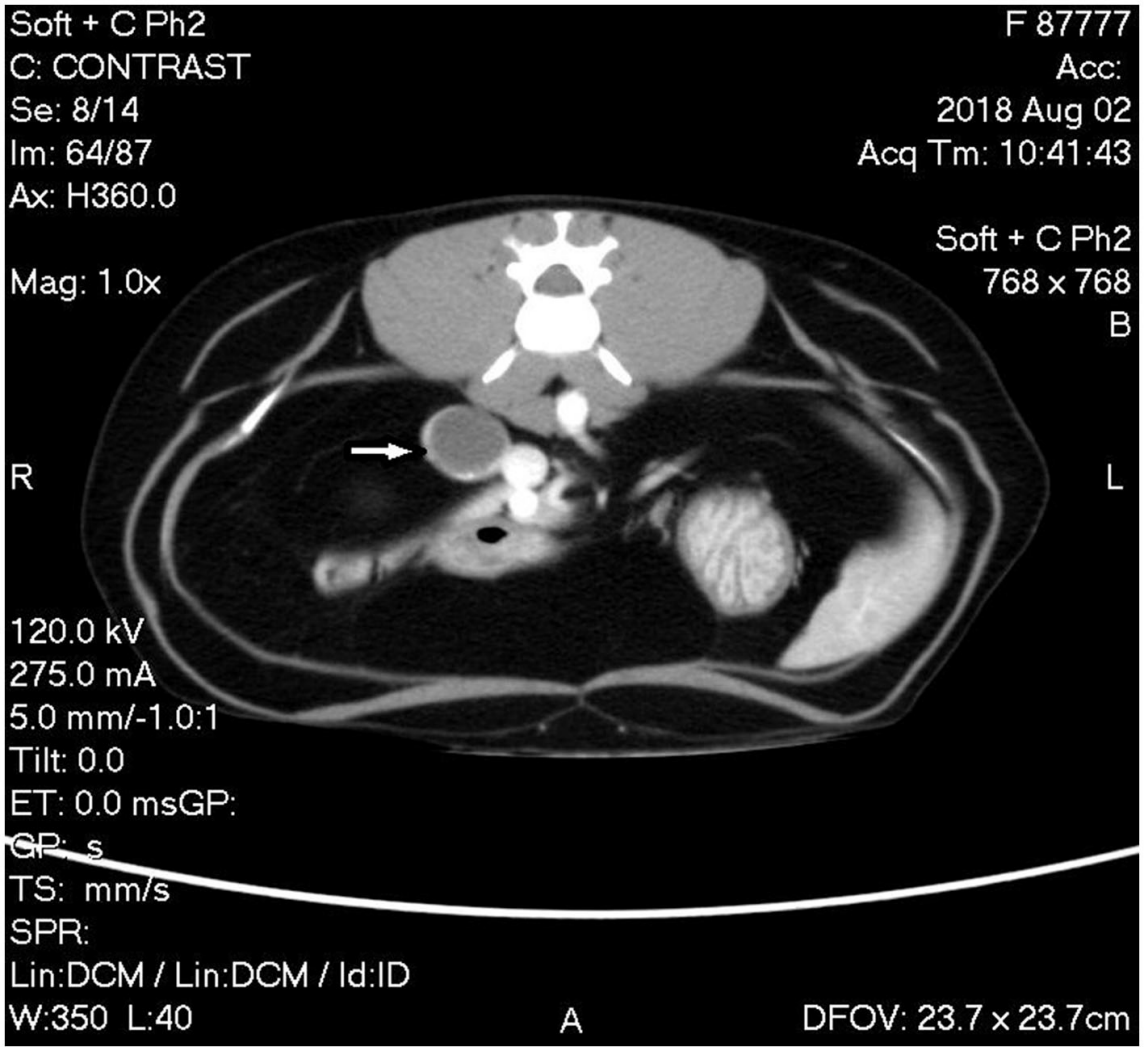
Computed tomography (CT) image of phase 2 post-contrast study of the abdomen showing the mass associated with the caudal pole of the right adrenal gland with rim enhancement and mild homogenous central enhancement (white arrow).

A triple phase-contrast abdominal computed tomography (CT) was performed to fully assess the adrenal mass and thoracic CT for metastasis evaluation. The cranial pole of the right adrenal was markedly enlarged at 1.8 cm diameter × 3.4 cm craniocaudal length with a hypoattenuating nodule in the caudal pole measuring 1.8 cm in diameter. The nodule had marked rim enhancement and mild homogenous central enhancement in the arterial and venous phases. The right phrenicoabdominal vein was in close apposition to the caudal aspect of the adrenal nodule and the right adrenal gland was in close apposition to the caudal vena cava without visible flattening or filling defects (Figure 2). The left adrenal gland was noted to be within normal limits at 0.6 cm dorsoventral diameter. There were multiple hypoattenuating splenic nodules, consistent with myelolipoma characteristics in the spleen of domestic cats.

The CT findings were consistent with a right adrenal mass without invasion into the caudal vena cava or renal vasculature. Differential diagnoses included possible neoplastic processes, such as adrenocortical adenoma and carcinoma or pheochromocytoma, although nodular hyperplasia could not be ruled out. Surgical exploration and right adrenal resection were recommended for excisional biopsy to determine the diagnosis.

### Therapeutic Intervention

**Figure 3:**
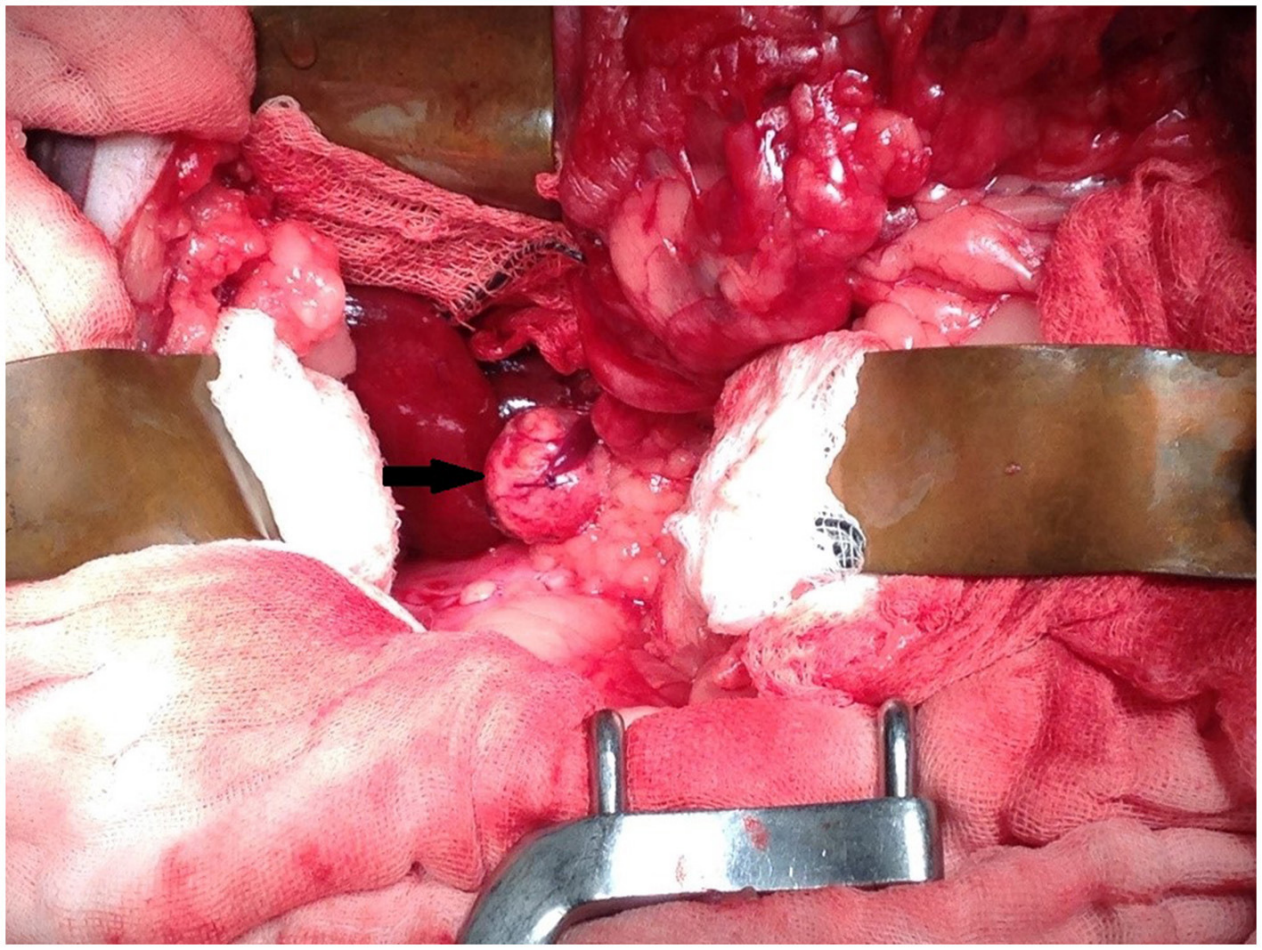
Intra-operative image showing mass associated with the right adrenal gland (arrow). Cranial is at the top of the image and caudal is at the bottom of the image.

Upon exploratory laparotomy *via* ventral midline celiotomy under general anesthetic, the previous enterotomy site was visualized in the proximal to mid jejunum with omental adhesions. The right kidney and adrenal gland were palpable, however, they could not be visualized due to abundant retroperitoneal fat. Malleable retractors were utilized to improve visualization and the overlying fat was carefully dissected following incision into the peritoneum overlying the right kidney until the adrenal gland and associated mass were visible (Figure 3). The caudal pole of the right adrenal gland was enlarged, firm, spherical in shape, and had a smooth capsule with no signs of invasion into the surrounding structures. On manipulation of the mass, the serval's blood pressure increased to 170 mmHg systolic (mean arterial 130 mmHg). The right phrenicoabdominal vein was identified, ligated, and transected both cranial and caudal to the adrenal mass. Once fully excised *en bloc* using sharp and blunt dissection, the adrenal gland and associated mass were submitted in formalin for histopathological examination. Once the mass had been removed, the serval's hypertension was resolved and remained stable for the remainder of the general anesthetic. A coagulation panel was performed immediately following surgery, and the platelet count was decreased; however, there was platelet clumping noted on the blood smear (automated count likely to be spuriously decreased due to clumping, the manual count was subjectively normal). Recovery from anesthesia was uneventful. The sample was submitted for special immunohistochemical staining with Chromogranin A, as the intraoperative hypertension raised the possibility of an unusual pheochromocytoma.

**Figure 4:**
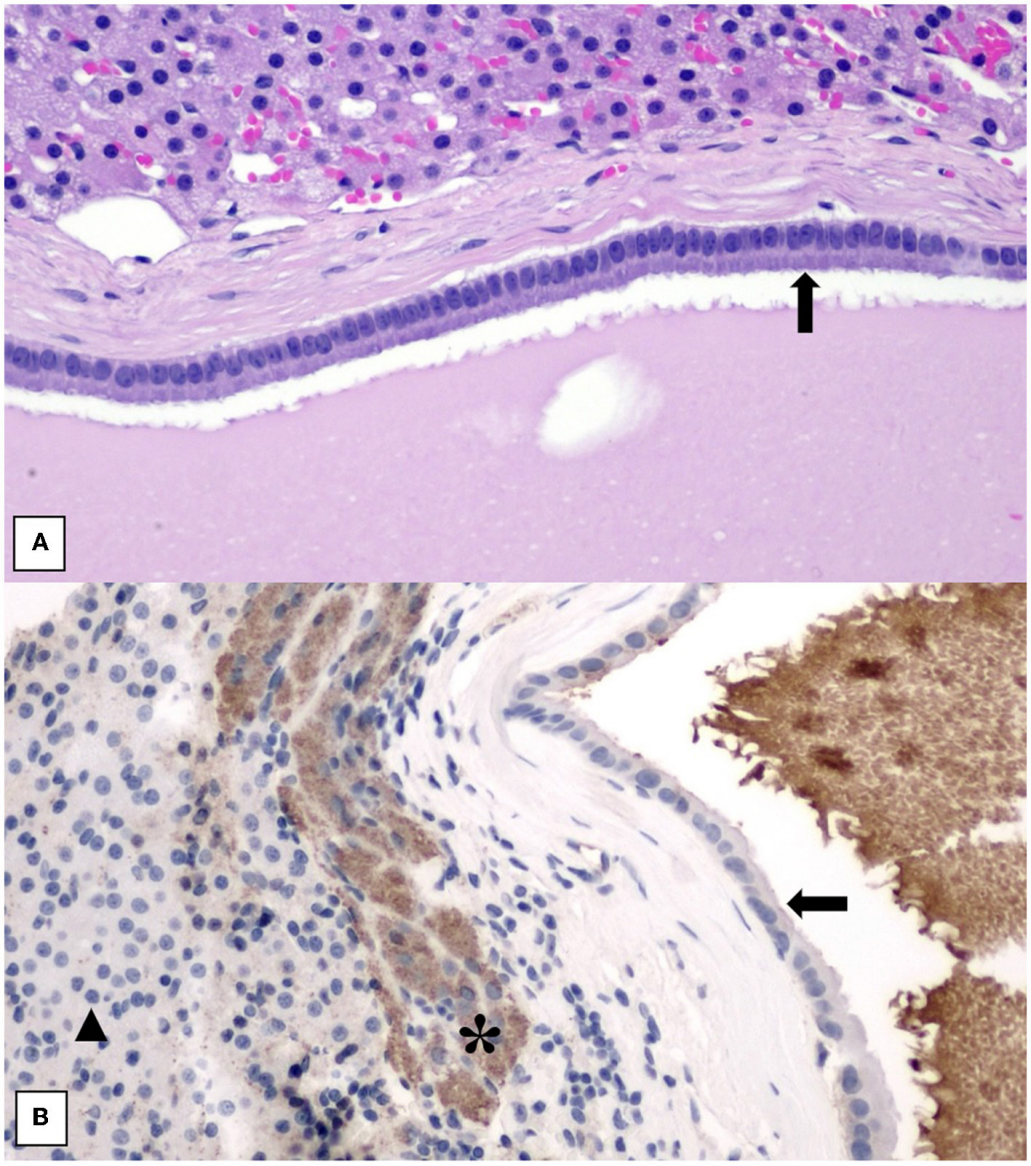
**(A)** Histology of adrenal cyst wall showing lining of uniform columnar epithelial cells (black arrow) overlying a thin fibrous capsule. Normal adrenal cortical parenchyma is at the top of the image. H&E stain. 200× magnification. **(B)** Histology of adrenal cyst wall stained with Chromogranin A showing negative staining of the cyst lining (black arrow) and non-specific staining of the proteinaceous cyst content. Internal positive control of adrenal medullary cells (*) and internal negative control of adrenal cortical cells (black triangle) is included. 200× magnification.

Histopathology showed an expansile, encapsulated cyst lined by a single layer of plump to attenuated to cuboidal to columnar epithelial cells (Figure 4). The cyst content comprised amorphous eosinophilic (proteinaceous) debris with cholesterol clefts, occasional macrophages, and multinucleated giant cells. Toward one edge of the cyst, there was hemorrhage and aggregates of mineralized debris. The adrenal cortex and medulla were compressed to a rim around the periphery of the cyst. These findings were consistent with an epithelial adrenal cyst. There was no evidence of neoplastic transformation. Due to intra-operative hypertension raising the possibility of an unusual pheochromocytoma, the sample was submitted for special immunohistochemical staining with Chromogranin A. Immunohistochemistry staining for Chromogranin A was performed, using an internal positive and a negative control consisting of areas of normal adrenal medullary and cortical parenchyma, respectively. The epithelial lining cells of the cyst were negative for Chromogranin A, which did not support a cyst of adrenal medullary epithelial origin/cystic pheochromocytoma. There was generalized indistinct staining of the proteinaceous cyst content, considered to be a non-specific reaction (Figure 4).

Since the surgery, the serval has been doing well, showing no clinical signs with a healthy appetite and normal activity.

## Discussion

Adrenal cysts are rare and have not been reported in a feline in the literature to the authors' knowledge. Other than compression atrophy of the adrenal parenchyma and potential for the rupture with associated inflammation, they are considered benign incidental findings.

An attempt has been made to categorize adrenal cysts in humans and the currently adopted method categorizes them into four main subtypes; epithelial, endothelial, pseudocysts, and parasitic hydatid cysts (1, 6, 7). An attempt has been made to further subdivide epithelial cysts based on their pathogenesis into retention cysts (embryonic malformation or inclusion of displaced urogenital tissue has been reported in humans), embryonal cysts, and cystic degeneration of adrenal cortical neoplasms (5, 9). It has been proposed that true adrenal epithelial cysts may be similar to epithelial cysts of the spleen in humans, consistent with a mesothelial origin by inclusion (10). Mesothelial markers, such as calretinin and Wilms tumor protein 1 (WT-1), would need to be performed to confirm this (9).

Adrenal cysts are also uncommonly reported in humans and therefore a consensus has not been reached in terms of management and treatment (1). Definitive diagnosis often cannot be reached without surgical excision and histopathology as clinical signs and imaging features are non-specific (1, 11). Cystic endocrine active adrenal tumors have been reported in the literature and therefore assessing for the potential functional status of an adrenal cystic lesion may be helpful when making a decision regarding surgical intervention (2). Blood and urine tests, such as resting serum cortisol concentration, low-dose dexamethasone suppression test, and urine metanephrine:norepinephrine ratios, can help to assess functional status, however, reference ranges for these tests are not readily available for exotic species. It is not unreasonable to opt to monitor a patient with no clinical signs and no features of malignancy on imaging in which an incidental adrenal mass was found; however, malignant masses can be mistaken for benign masses on imaging and the consequences of conservative monitoring in these cases may be detrimental to the patient (1, 11). There is currently no protocol for surveillance of such lesions in humans or small animals.

In humans, some authors have recommended surgical excision of all symptomatic functional cysts, cysts >5 cm in size due to risk of hemorrhage, and cysts with a hemorrhagic or heterogeneous nature on imaging suggestive of malignancy (2–4). Percutaneous fine-needle aspiration (FNA) can be performed and has been shown in a study in the human literature to have a sensitivity of 85% for detecting malignancy and for correctly classifying adrenal masses in 90% of cases (2, 5). An FNA should always be accompanied by a cystogram. If there is hemorrhagic fluid on aspiration, cytology suggestive of malignancy, or an irregular cyst lining on cystogram, the current recommendation in human medicine is to then move to complete surgical excision (6, 7). Marsupialization has been described as an option in humans with large adrenal cysts, which are adhered to multiple organs making excision difficult (8). For both FNA and marsupialization, the risks of seeding into the abdominal cavity due to cyst leakage have not been evaluated but would be a potential concern for these procedures. Surgical excision is always the recommended treatment for a parasitic hydatid cyst, a functioning or a malignant cyst and many surgeons are now performing surgical excisions in humans laparoscopically with much lower morbidities (2, 12).

The blood pressure of this patient was increased during the surgical manipulation of the adrenal epithelial cyst. Intra-operative hypertension is often seen during surgical removal of pheochromocytomas due to surges in catecholamine concentration in the blood (13). This intra-operative finding prompted the histopathological chromogranin A staining to assess for a potential pheochromocytoma. It is unknown why this episode of intra-operative hypertension was encountered but possible causes may include intra-operative pain or catecholamine releases from the normal adrenal tissue surrounding the cyst.

Limitations of the case reported in this article include limited conscious examination of the animal due to its fractious nature and reporting on the surgical intervention and outcome of a single patient. However, having repeating ultrasound examinations followed by a triple phase-contrast CT scan of the abdomen allowed a thorough depiction of the adrenal epithelial cyst and histopathology of the excised mass with the inclusion of special stains allowed for a definitive novel diagnosis.

In conclusion, benign adrenal cysts should be included on the differential list for an enlarged adrenal gland or an adrenal mass found in imaging studies. Current treatment and monitoring recommendations must be extrapolated from the human literature as there have not been adrenal cysts previously described in the veterinary literature for felines. As adrenal cysts are rare in humans, controversy still exists as to the best treatment option. Due to the non-specific clinical signs and imaging characteristics of cysts, surgical excision should be considered for all masses as this will allow a definitive pathological diagnosis and prevent interpretation of a malignant tumor on imaging as a benign incidentaloma (14).

## Data Availability Statement

The raw data supporting the conclusions of this article will be made available by the authors, without undue reservation.

## Ethics Statement

Ethical review and approval was not required for the animal study because this study was a retrospective analysis of the medical records from the School of Veterinary Science and therefore did not require approval by an Animal Ethics Committee. Written informed consent was obtained from the owners for the participation of their animal in this study.

## Author Contributions

SD drafting of manuscript and literature review. WB editing of manuscript. SP histopathological analysis and drafting summary of findings for this article. MO imaging interpretation and drafting summary of findings for this article. All authors contributed to the article and approved the submitted version.

## Conflict of Interest

The authors declare that the research was conducted in the absence of any commercial or financial relationships that could be construed as a potential conflict of interest.

## Publisher's Note

All claims expressed in this article are solely those of the authors and do not necessarily represent those of their affiliated organizations, or those of the publisher, the editors and the reviewers. Any product that may be evaluated in this article, or claim that may be made by its manufacturer, is not guaranteed or endorsed by the publisher.
